# Gel Formulations with an Echinocandin for Cutaneous Candidiasis: The Influence of Azone and Transcutol on Biopharmaceutical Features

**DOI:** 10.3390/gels9040308

**Published:** 2023-04-06

**Authors:** Noelia Pérez-González, Lupe Carolina Espinoza, María Rincón, Lilian Sosa, Mireia Mallandrich, Joaquim Suñer-Carbó, Nuria Bozal-de Febrer, Ana Cristina Calpena, Beatriz Clares-Naveros

**Affiliations:** 1 Department of Pharmacy and Pharmaceutical Technology, Faculty of Pharmacy, University Campus of Cartuja, University of Granada, 18071 Granada, Spain; 2 Departamento de Química, Universidad Técnica Particular de Loja, Loja 1101608, Ecuador; 3 Departament de Ciència de Materials i Química Física, Facultat de Química, Universitat de Barcelona (UB), C. Martí i Franquès 1-11, 08028 Barcelona, Spain; 4 Institut de Nanociència i Nanotecnologia (IN2UB), Universitat de Barcelona (UB), 08028 Barcelona, Spain; 5 Pharmaceutical Technology Research Group, Faculty of Chemical Sciences and Pharmacy, National Autonomous University of Honduras (UNAH), Tegucigalpa 11101, Honduras; 6 Departament de Farmàcia, Tecnologia Farmacèutica, i Fisicoquímica, Facultat de Farmàcia i Ciències de l’Alimentació, Universitat de Barcelona (UB), 08028 Barcelona, Spain; 7 Departament de Biologia, Sanitat i Medi Ambient, Facultat de Farmàcia i Ciències de l’Alimentació, Universitat de Barcelona (UB), 08028 Barcelona, Spain; 8 Biosanitary Institute of Granada (ibs.GRANADA), 18012 Granada, Spain

**Keywords:** caspofungin, echinocandin, Azone, candidiasis, permeation enhancer, gel

## Abstract

Caspofungin is a drug that is used for fungal infections that are difficult to treat, including invasive aspergillosis and candidemia, as well as other forms of invasive candidiasis. The aim of this study was to incorporate Azone in a caspofungin gel (CPF-AZ-gel) and compare it with a promoter-free caspofungin gel (CPF-gel). An in vitro release study using a polytetrafluoroethylene membrane and ex vivo permeation into human skin was adopted. The tolerability properties were confirmed by histological analysis, and an evaluation of the biomechanical properties of the skin was undertaken. Antimicrobial efficacy was determined against *Candida albicans*, *Candida glabrata*, *Candida parapsilosis*, and *Candida tropicalis*. CPF-AZ-gel and CPF-gel, which had a homogeneous appearance, pseudoplastic behavior, and high spreadability, were obtained. The biopharmaceutical studies confirmed that caspofungin was released following a one-phase exponential association model and the CPF-AZ gel showed a higher release. The CPF-AZ gel showed higher retention of caspofungin in the skin while limiting the diffusion of the drug to the receptor fluid. Both formulations were well-tolerated in the histological sections, as well as after their topical application in the skin. These formulations inhibited the growth of *C. glabrata*, *C. parapsilosis*, and *C. tropicalis*, while *C. albicans* showed resistance. In summary, dermal treatment with caspofungin could be used as a promising therapy for cutaneous candidiasis in patients that are refractory or intolerant to conventional antifungal agents.

## 1. Introduction

Candidiasis is a fungal infection caused by the yeast of the genera *Candida* spp., which is the most important cause of opportunistic mycoses worldwide and can proliferate in the skin and mucosal surface, even causing a systemic infection [1,2]. *Candida albicans* is the most prevalent pathogen that is responsible for about 70% of fungal infections [2]. Cutaneous candidiasis is caused by *Candida* proliferation in the skin, mainly in the intertriginous areas, producing inflammation, dryness, erosions, and pustules [3,4]. Despite the available pharmacological treatments, such as azoles and polyenes, complications from *Candida* infections are frequent, especially in patients that are refractory or intolerant to these antifungal drugs or those in hospital conditions, including immunosuppressed populations such as patients with malignancies or HIV infections for whom the mortality rates are high [4,5].

**Figure 1:**
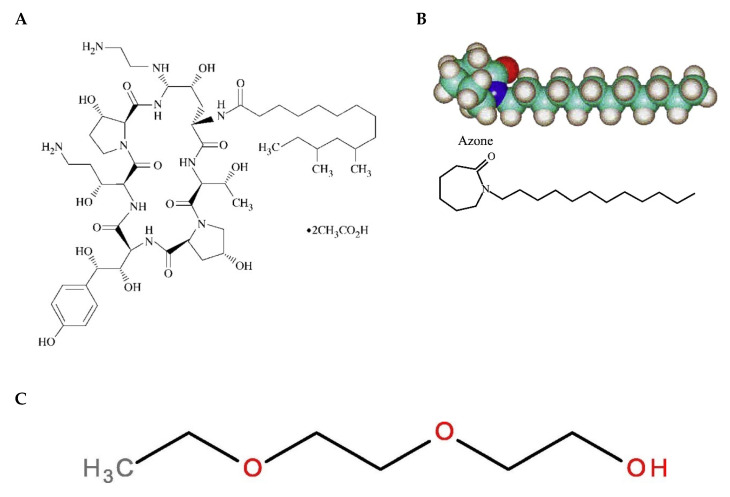
Chemical structure of drug and skin promoters. (**A**) Caspofungin [27], (**B**) Azone, [20] and (**C**) diethylene glycol monoethyl ether (Transcutol) [28].

Therefore, the treatment of candidiasis becomes a challenge in this population, and consequently, other pharmacological options, such as echinocandins, have been addressed [6]. Echinocandin drugs are formed by a cyclic nucleus composed of several amino acid residues and N-linked acyl lipophilic fatty acid tails, which act as an anchor for the drug at the pathogen cell wall. These antifungal drugs inhibit the biosynthesis of β-(1,3)-D-glucan, which is present in the fungal cell walls, including in Aspergillus and *Candida* spp. [5,7,8]. Currently, there are some clinically used echinocandins, such as caspofungin, micafungin, and anidulafungin, all of which have a lipopeptide structure that is synthetically modified from the fermentation broths of different fungi [8]. Caspofungin is a semi-synthetic lipopeptide obtained from a fermentation product of *Glarea lozoyensis* [9]; its chemical structure is depicted in Figure 1. In 2001, caspofungin was approved by the US Food and Drug Administration (FDA) and the European Medicines Agency (EMEA). This drug is used as therapy for fungal infections that are difficult to treat, including invasive aspergillosis, as well as candidemia and other forms of invasive candidiasis [6,10]. However, caspofungin is only used for intravenous administration due to its high molecular weight (1093.31 g/mol) and poor oral bioavailability, and consequently, the need arises to investigate alternative formulations and routes of administration to improve the efficacy of the drug and facilitate its use [9]. Dermal treatment with caspofungin could be used as a promising therapy for cutaneous candidiasis in patients that are refractory or intolerant to conventional antifungal agents.

In recent years, formulations combining polymers have been designed to produce multifunctional gels [11]. The combination of polymers of a natural origin, such as chitosan, with polymers of a synthetic origin, such as poloxamer 407, produces gels with a biocompatible and biodegradable nature, with a high permeability capacity and, thus, better therapeutic effect [12]. Chitosan is a cationic polysaccharide consisting of glucosamine and N-acetylglucosamine units obtained by the alkaline deacetylation of the natural polysaccharide chitin and is known for its antimicrobial properties. It is biocompatible, biodegradable, and capable of forming gels in situ with negatively charged macromolecules [13,14,15]. On the other hand, poloxamer 407 is a hydrophilic nonionic surfactant consisting of a hydrophobic polypropylene oxide (PPO) core block flanked by hydrophilic poly (ethylene oxide) (PEO-PPO-PEO) blocks and is known to be thermoreversible [16]. To that end, skin penetration promoters can be used in order to facilitate the drug permeation through the stratum corneum and achieve an effective drug concentration [17]. Several permeation enhancers, including pyrrolidones, Azone, alcohols, sulfoxides, surfactants, essential oils, and their chemical constituents, have been proposed in cosmetic and pharmaceutical formulations to improve transdermal drug delivery and increase drug retention in the skin [18,19]. The ideal purpose of these permeation enhancers should be to temporarily and reversibly perturb the barrier function of the stratum corneum [19]. The reported mechanisms of action for these skin penetration promoters include the (i) reversible alteration of the intercellular lipid matrix or on the intracellular keratin domains, (ii) changes in the drug/tissue partition coefficient, and (iii) the disturbance of skin metabolism [20]. Azone (1-dodecylazacycloheptan-2-one) was the first synthetic compound specifically designed as a skin permeation promoter [21]. The chemical structure of Azone (Figure 1) shows a polar headgroup (within a seven-membered ring) attached to a C_12_ chain, which possibly allows this molecule to interact directly with the skin lipid domains to disturb the organized lipid packing [19,22]. Transcutol-P (diethylene glycol monoethyl ether) is a compound that is extensively used as a penetration enhancer in topical delivery systems, as well as for its solubilizing action [23,24,25,26]; Figure 1 depicts its chemical structure.

**Figure 2:**
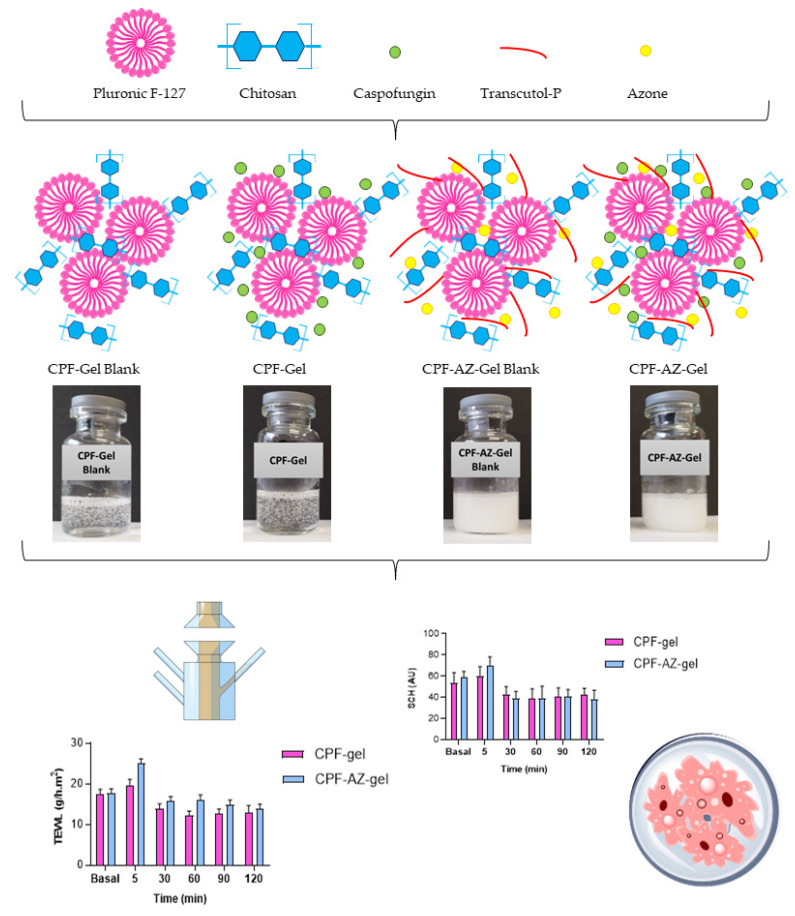
Schematic representation of the development of the loaded and unloaded hydrogels composed of pluronic F-127 and chitosan, with and without permeation enhancers, and the evaluation of the formulations.

Considering these remarkable findings, the aim of the study was to design and develop a topical delivery system for caspofungin. Two formulations were developed: a caspofungin gel (CPF-gel) and a caspofungin gel with the addition of two promoters: Azone and Transcutol-P (CPF-AZ-gel). The physicochemical, biopharmaceutical, and efficacy properties of both formulations in the treatment of cutaneous candidiasis were compared. Figure 2 illustrates a schematic representation of the development and evaluation of the hydrogels composed of pluronic F-127 and chitosan, with and without permeation enhancers loading caspofungin.

## 2. Results and Discussion

### 2.1. Preparation of Gel Formulations

**Figure 3:**
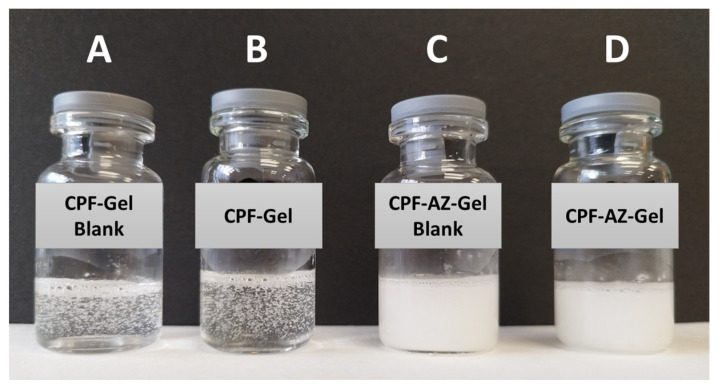
Photograph of drug-loaded hydrogels and nondrug-loaded hydrogels. (**A**) CPF-Gel without drug; (**B**) CPF-Gel; (**C**) CPF-AZ-Gel without drug, and (**D**) CPF-AZ-Gel.

The composition of CPF-gel and CPF-AZ-gel obtained in this work are described in Table 1. CPF-gel was obtained by dissolving Pluronic F-127 (18%) and caspofungin (2%) in cold water (58%), then chitosan (2%) that was previously dissolved in 0.1 M aqueous acid acetic solution (20%) was added. On the other hand, CPF-AZ-gel was obtained by incorporating a mixture of Azone (5%) and Transcutol-P (5%) into CPF-gel that was previously prepared by considering water adjustment. Both gels (CPF-gel and CPF-AZ-gel) had a uniform and homogeneous appearance, free of visible particles, lumps, and precipitates (Figure 3).

**Figure 4:**
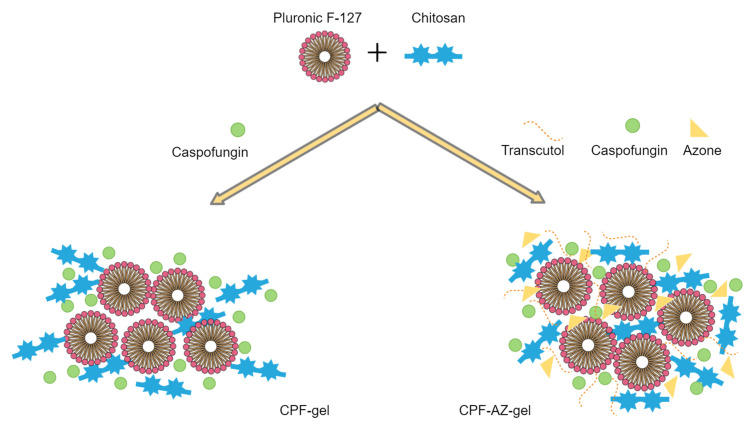
Schematic illustration of the mechanism of hydrogel formation.

The gelling of CPF-gel and CPF-AZ-gel occurs because of the Pluronic F-127 and chitosan content. In an aqueous solution, the amphiphilic properties of Pluronic F-127 cause self-aggregation-forming micelles at concentrations above its critical micelle concentration, where PPO hydrophobic groups interact together via van der Waals forces to form the inner core, whereas the PEO hydrophilic groups interact with water molecules via hydrogen bonds to form the outer shell [29,30]. Consequently, gelation occurs at sufficient concentrations of Pluronic F-127, which, in this study, was 18% due to micelles packing [31]. On the other hand, the gelling with chitosan was produced by physical cross-linking, where ion interactions and hydrogen bonds are the driving forces for the formation of the entangled networks of this gel [32,33]. Figure 4 illustrates the mechanism of hydrogel formation.

### 2.2. Thermosensitive Properties

**Figure 5:**
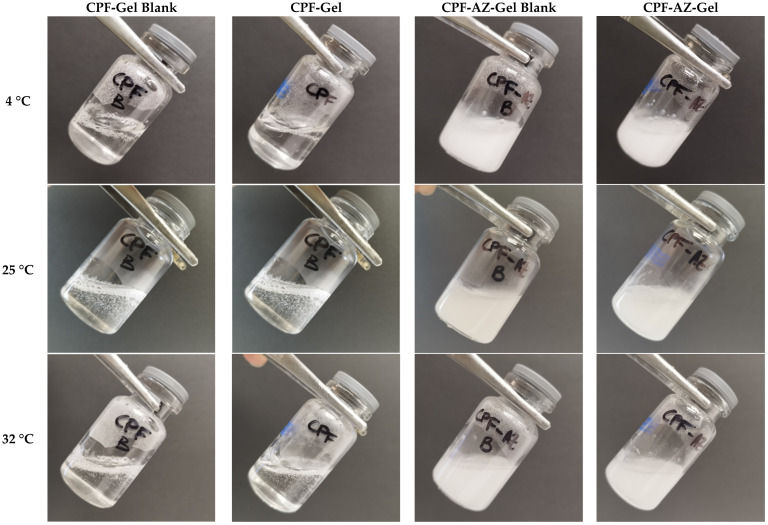
Physical appearance of the hydrogels at different temperatures: 4 °C, 25 °C, and 32 °C.

The incorporation of the drug into the hydrogels did not change the temperature sensitivity, as shown in Figure 5. No significant differences were observed when the gels were acclimatized to the different temperatures after the addition of the Azone and Transcutol-P promoters. As the temperature increased, the gels seemed to flow better. However, at no time was the gelation state reached.

Combinations of polymers, such as CTS and P407, can modify the properties of the resulting system. The micellar corona increases in thickness because the number of hydrophilic chains increases. This is due to the interaction between CTS and the polyethylene oxide chains of P407. As a result of the interaction, the apparent viscosity increases, leading to a decrease in temperature, whereby the SOL-GEL transition could be reached [34,35].

### 2.3. Fourier Transform Infrared

**Figure 6:**
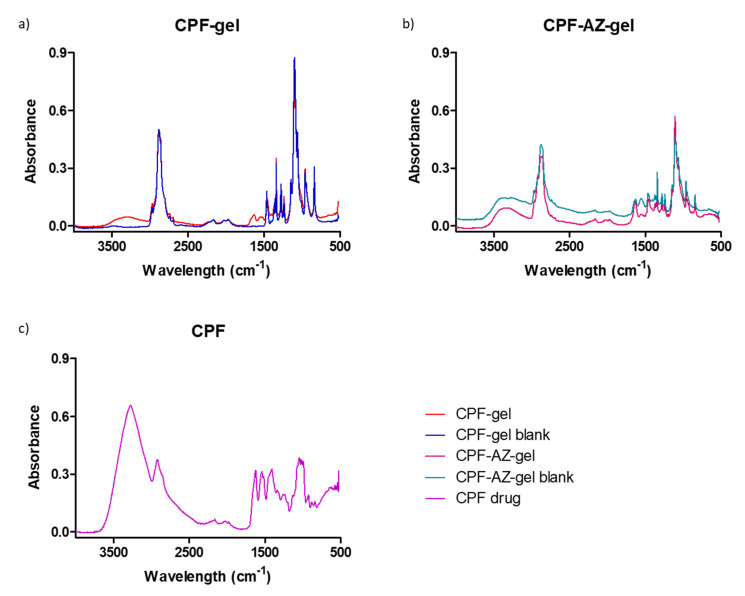
FTIR spectra for the gels: (**a**) CPF gel (red) and placebo (blue), (**b**) CPF-AZ-gel (pink) and placebo (cyan), and (**c**) caspofungin acetate salt Sun-Pharma (purple).

Fourier transform infrared (FTIR) was performed to investigate any possible interaction between the drug and the gel matrix. Figure 6 shows the FTIR spectra for the loaded and unloaded gels of CPF-gel and CPF-AZ-gel. The peaks corresponding to different functional groups of caspofungin can be seen at about 1600 cm^−1^: carbonyl and amide. Within the range 3200–3600 cm^−1^, there are peaks related to O-H bond vibrations from the alcohol groups present in the structure, as well as from the amide groups (Figure 6c). When analyzing the hydrogels loaded with caspofungin, a remarkable decrease in the absorption is observed within the range 3000–3600 cm^−1^ (OH stretching) compared to the caspofungin spectrum. This fact indicates that caspofungin interacts with the hydrogel via the hydrogen bonds, especially between the hydroxyl groups in both caspofungin and the polymers.

### 2.4. Morphological Study and Determination of the Porosity of the Hydrogels

**Figure 7:**
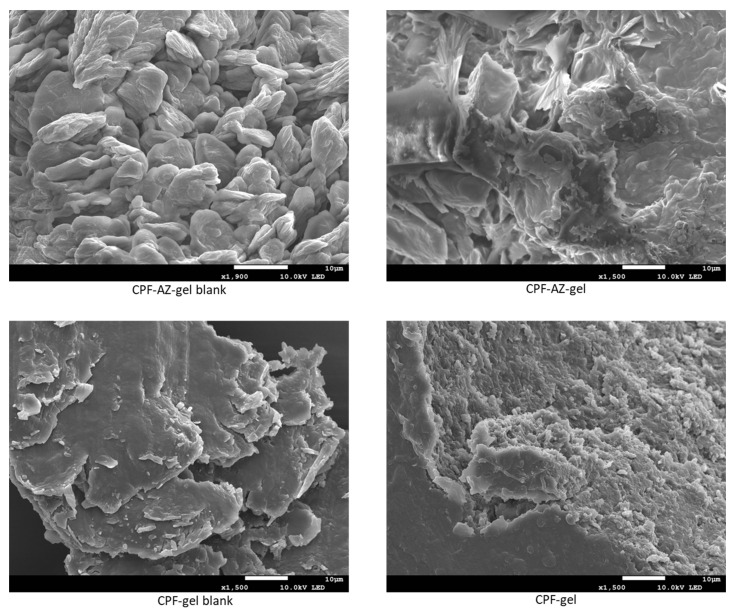
SEM images of the hydrogels: left panels show the blank hydrogels with or without permeation enhancers, and the right panels display the loaded hydrogels.

The structure of the gels was evaluated via scanning electron microscopy (SEM) after desiccating the gels. Figure 7 shows the SEM images of the unloaded and loaded hydrogels. CPF-gel showed a denser and more compact structure than CPF-AZ-gel. The addition of Transcutol-P and Azone resulted in a more rounded shape structure, which was especially remarkable for the unloaded gels.

Hydrogels have the capacity to absorb large amounts of water or biological fluids; when the gels absorb more than 10 times their weight (in the dried state), they are classified as superabsorbent hydrogels. There are different techniques to evaluate gel porosity; in this work, the porosity was determined by the density of the solvent uptake. CPF-AZ-gel showed higher porosity than CPF-gel (Table 2). The addition of Transcutol-P and Azone in the hydrogel increased the porosity by about four-fold. The porosity of the gels has an impact on the drug release from the gel matrix [36]. This is in line with the drug release behavior observed in Section 2.7; CPF-AZ-gel released greater amounts of caspofungin than CPF-gel.

### 2.5. Rheological Behavior

**Figure 8:**
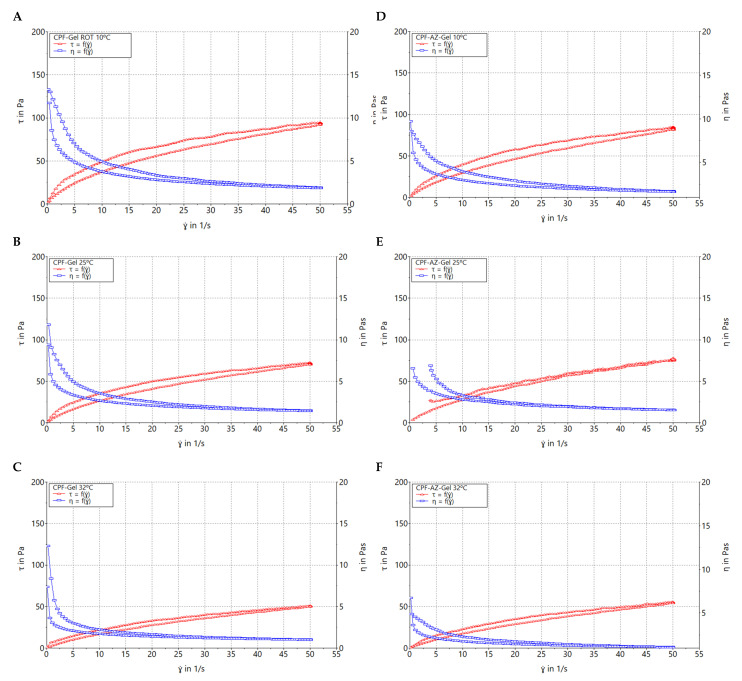
Rheograms of caspofungin formulations. (**A**) CPF-gel at 10 °C, (**B**) CPF-gel at 25 °C, (**C**) CPF-gel at 32 °C, (**D**) CPF-AZ-gel at 10 °C, (**E**) CPF-AZ-gel at 25 °C, and (**F**) CPF-AZ-gel at 32 °C.

Figure 8 shows the rheograms of CPF-gel and CPF-AZ-gel at 10, 25, and 32 °C. Rheological behavior plays a critical role in topical formulations, as it determines sensory and dosage characteristics, as well as modulates the biopharmaceutical parameters, such as the drug release rate from its vehicle [37]. CPF-AZ-gel exhibited a higher viscosity than CPF-gel due to its Azone and Transcutol-P content, providing greater consistency to the final product. The viscosity of both formulations decreased as the temperature increased, showing values of 1.48, 1.16, and 0.95 Pa·s at 10, 25, and 32 °C, respectively, for CPF-gel and 1.66, 1.52, and 1.09 Pa·s at 10, 25, and 32 °C, respectively, for CPF-AZ-gel.

Table 3 summarizes the results obtained from the rheological analysis of both formulations and the mathematical modeling of the experimental data. Both CPF-gel and CPF-AZ-gel at 10, 25, and 32 °C exhibit shear-thinning (pseudoplastic) behavior, according to the Cross model for the stretch ramp-up and stretch ramp-down. This behavior is characterized by a decrease in viscosity as friction or light massage is applied to the treated area facilitating its spreadability on the skin; however, when the friction is stopped, the viscosity of the product returns to its original state, favoring the product residence time [38].

### 2.6. Spreadability Analysis

**Figure 9:**
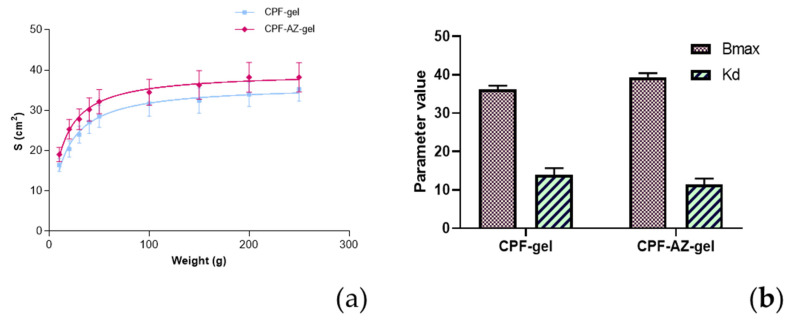
Evaluation of the spreadability of CPF-gel and CPF-AZ-gel, fitting a one-site hyperbola equation: (**a**) Spreading area (S, cm^2^) as a function of the applied mass (g) at 22 ± 2 °C; 60 ± 5% RH, and (**b**) plot of the parameters of the formulas of CPF-gel, and CPF-AZ-gel fitting a one-site hyperbola equation. The data are expressed as the mean ± standard deviation (SD) of the three replicates (n = 3).

Sample spreading properties represent decisive parameters in the assessment of topical forms as they affect the process of incorporation into the container as well as the uniformity of the dose and application, thereby affecting therapeutic efficacy [39,40]. CPF-gel and CPF-AZ-gel followed a hyperbola one-site model (Figure 9), and similar values were observed regarding the extensibility of the two formulas (Table 4). The results obtained indicate that both formulas can be easily spread on the skin.

### 2.7. Evaluation of the Drug-Release Kinetics

The amount of caspofungin that was released from the formulations was assessed by in vitro release tests performed by Franz cells. A suitable receptor medium for the in vitro drug release studies must provide sink conditions so as to ensure that the concentration of the drug in the receiver compartment will not exceed 30% of the concentration to the saturation of the given drug in that medium [41]. Keeping sink conditions is crucial and prevents the inhibition of drug diffusion through the membrane/skin due to the saturation of the medium [42]. In this study, 0.9% physiological saline solution provided the sink conditions for the study.

**Figure 10:**
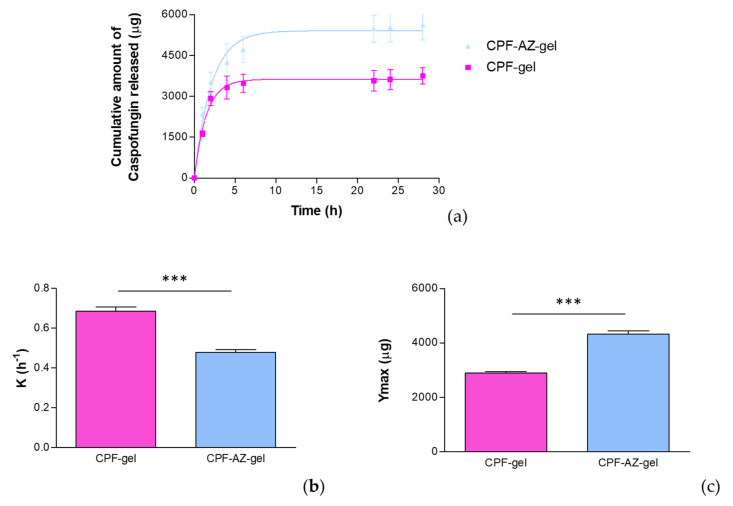
In vitro drug release of caspofungin from the CPF-gel and CPF-ZA gel formulations: (**a**) release profile of caspofungin over time; (**b**) release constant (K), estimated by modeling to the best-fit model, corresponding to a one-phase exponential association model; (**c**) maximum amount released (Y_max_) estimated by modeling to the best-fit model, corresponding to a one-phase exponential association model. *** significant statistical differences between the two gels (*p* < 0.0001).

Figure 10 shows the release profile of caspofungin using a polytetrafluoroethylene (PTFE) membrane. The selection of the membrane in the drug-release studies is also crucial since they must be inert and must not limit the drug release, nor can any binding between the drug and the membrane occur [41].

The release data were fitted to the kinetic models to describe the release profile of caspofungin. The best fit was obtained for a one-phase exponential association model (r^2^ = 0.9986 for CPF-gel and r^2^ = 0.9971 for CPF-AZ-gel), for which the mathematical equation is

(1) $$Y=Y_{max}\cdot \left(1-e^{-K\cdot X}\right)$$

where *K* is the release rate, *Y_max_* is the maximum amount of drug released, and *X* is the time. In this study, the parameters *K* and *Y_max_* were estimated and statistically compared by a *t*-test. According to the one-phase exponential association kinetics, the release of caspofungin is directly proportional to the concentration of the drug remaining in the donor chamber, resulting in a fast drug release in the early stages when the concentration of the drug is high in the donor chamber, and followed by a slowing down of the release rate as the concentration in the donor chamber decreases.

Although CPF-gel showed a greater release rate, it resulted in lower amounts of drug released than CPF-AZ-gel, which suggests that Azone and Transcutol-P improve the availability of the drug, and higher amounts of caspofungin would be delivered on the skin from the CPF-AZ-gel formulation.

### 2.8. Evaluation of the Permeation Capacity of Caspofungin through Ex Vivo Human Skin

The capacity of caspofungin to diffuse through the skin was evaluated by an ex vivo permeation study. In the ex vivo permeation studies, keeping sink conditions correct is as essential as in the in vitro drug release study. Furthermore, the receptor fluid, apart from providing sink conditions, must be biocompatible with the skin; for this reason, a 0.9% physiological saline solution was selected. Another important critical factor in permeation studies is the integrity of the ex vivo skin because using impaired skin or skin with altered barrier functions affects the permeability results. Hence, it is important to assess the skin integrity of the discs included in the study. Transepidermal water loss (TEWL) is one technique to evaluate skin integrity since there is a high correlation between TEWL values and skin integrity. Dey and co-workers investigated the TEWL values of intact skin, and then the researchers damaged the skin at different severities by removing stratum corneum with tape strips. They observed that the TEWL values for intact skin were below 13 g/m^2^/h; moderately compromised skin was prepared with 20 consecutive tape strips and showed TEWL values up to 15.6 g/m^2^/h; a total of 25–30 tape strips corresponded to highly compromised skin, for which the TEWL values increased up to 35 g/m^2^/h. The maximum damage was reached by separating the epidermis from the dermis with heat treatment; this extremely damaged skin resulted in TEWL values up to 44.8 g/m^2^/h [43]. The stratum corneum is the outermost layer of the skin and is the main factor responsible for the barrier function. Skins with altered barrier functions are usually more permeable than intact skins. For this reason, the integrity of the skin discs used in this study was considered, and only those with TEWL values below 13 g/m^2^/h were included in the study.

**Figure 11:**
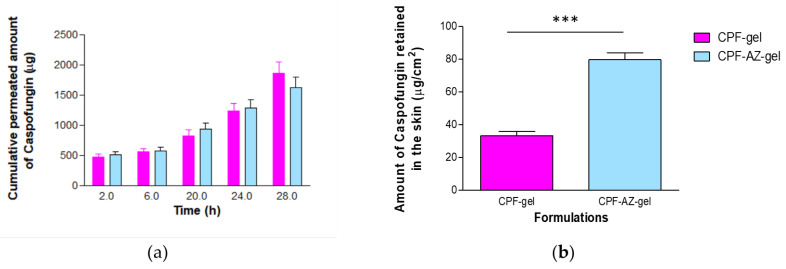
Permeation of caspofungin from CPF-gel and CPF-AZ-gel: (**a**) permeation profiles of caspofungin depicted as the cumulative amounts of caspofungin permeated through the ex vivo skin over time; (**b**) amount of caspofungin remaining in the skin at the end of the permeation study (28 h). *** significant statistical differences between the two gels (*p* < 0.0001).

Figure 11 shows the amounts of caspofungin permeated over 28 h and those retained in the skin at the end of the experiment.

Similar amounts of caspofungin permeated through the human skin for most of the sampling time points; contrarily, the formulation CPF-AZ-gel led to a major drug retention within the skin at 28 h. This is probably due to the presence of Azone and Transcutol-P in the formulation. Azone is known to be a penetration enhancer that is useful for increasing the permeation of both hydrophilic and lipophilic drugs. Azone increases the fluidity of the skin lipids by disrupting the lipid bilayers of the skin [44]. Transcutol-P is widely used in topical and oral formulations to improve the solubility of poorly soluble drugs; it disrupts the stratum corneum of the skin [45,46].

The permeation parameters, such as the flux and the cumulative amount of drug permeated at a given time, characterize the rate and extent of the drug through the skin. Evaluating these parameters is important in the development and optimization of topical products. Table 5 reports typical permeation parameters.

The amount of caspofungin that permeated through the skin over 28 h was slightly higher from the CPF-gel; however, no statistical differences were observed between the formulations for this parameter. The flux corresponds to the permeation rate of the drug through the skin, which is obtained as the slope of the linear regression of the linear part of the permeation profile; this parameter can also be expressed per unit area. The flux was 1.5-fold higher for CPF-gel, indicating that caspofungin crosses the skin faster when applied formulated in CPF-gel than CPF-AZ-gel. Apparently, the inclusion of Azone and Transcutol-P restricts the permeation of caspofungin and retains the drug in the skin. This is an interesting result for local therapy. The same pattern was observed for parameters Tl and K_P_, which were 1.5-fold higher for CPF-gel with respect to CPF-AZ-gel. A shorter lag time predicts an earlier onset of action; hence, it is expected that CPF-AZ-gel will initiate its therapeutical action more rapidly than CPF-gel. The permeability coefficient is greater for CPF-gel, which is logical because K_P_ is estimated from the flux, and CPF-gel exhibited a higher flux. The higher the permeability coefficient, the higher the permeation of the drug into the receptor fluid.

The permeation of drugs depends on both the partition and diffusion processes. When analyzing the partition and diffusion coefficients, it is evident that the partition has a greater impact on the permeation of caspofungin since it shows higher values than the diffusion coefficient. Interestingly, the addition of Azone and Transcutol-P increases the partition coefficient of caspofungin between the formulation and the skin with regard to the gel without the enhancers while decreasing the diffusion of caspofungin through the skin, resulting in a higher retention of caspofungin in the skin. This is also observed when analyzing the predicted plasma concentration at the steady state (C_ss_); CPF-AZ-gel would reach a lower C_ss_ than CPF-gel. C_ss_ was estimated by taking into account the human plasma clearance for caspofungin (9.42 L/h) [19] and considering an application surface of 5 cm^2^.

### 2.9. Histological Analysis of the Ex Vivo Human Skin after Permeation of Caspofungin

**Figure 12:**
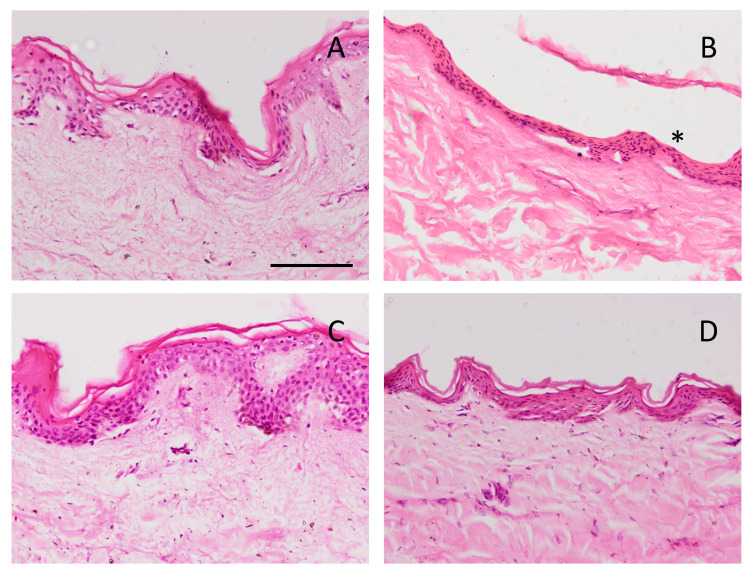
Skin sections stained with hematoxylin and eosin after the ex vivo permeation study, stained and observed under the microscope at 200X. Scale bar = 100 µm. (**A**) PBS as negative control; (**B**) ethanol as positive control; (**C**) CPF-gel and (**D**) CPF-AZ-gel. Asterisk indicates loss of stratum corneum.

After the ex vivo permeation test, in which we evaluated the permeability capacity of caspofungin from the two gels, an histological analysis was carried out to assess whether any structural change on the skin occurred. Figure 12 shows the histological images of the formulations, as well as the positive and negative controls. The ethanol solution induced the loss of stratum corneum. CPF-gel and CPF-AZ-gel did not change the stratum corneum or the epithelium.

### 2.10. Tolerance Studies by Evaluating Biomechanical Skin Properties

The skin integrity indicates the state of the skin as a physical barrier that protects the body from the environment. The integrity of the skin can be evaluated by measuring TEWL, which determines the ability of the skin to prevent water loss. TEWL increases when the skin barrier is compromised, for instance, via a cut, burn, or some skin diseases, including atopic dermatitis, eczema, or psoriasis. Altered skin barrier function, which may lead to increased transepidermal water loss, can result in irritation and skin dryness. Other factors can also impact the TEWL values, such as age and gender. For example, aging skin typically has a higher TEWL than younger skin, and exposure to dry or hot environments can increase TEWL.

Hence, measuring TEWL can be useful in dermatology and cosmetic science to evaluate the effectiveness of skin care products and treatments, as well as in clinical research to assess the skin barrier function in individuals with skin diseases or conditions [47].

**Figure 13:**
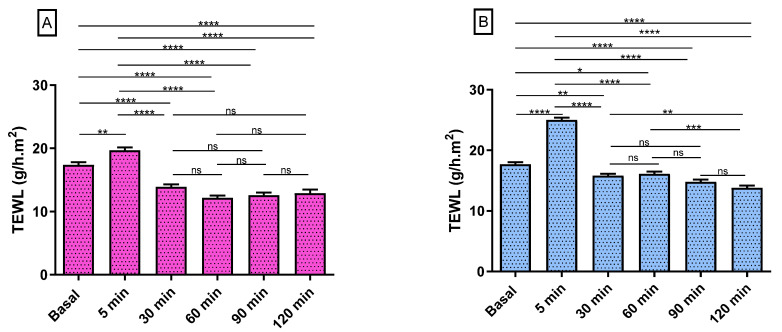

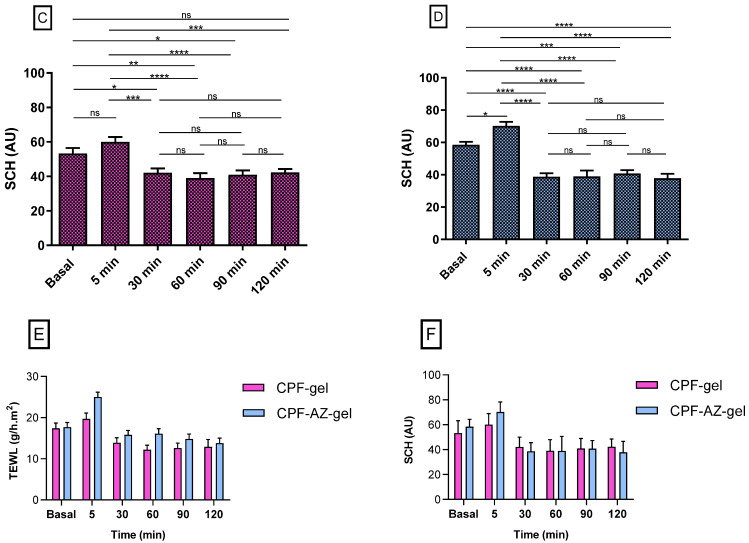
Biomechanical parameters evolution in human volunteers was monitored before the application of the formulations (basal) and 5 min, 30 min, 60 min, 90 min, and 120 min post-application. (**A**,**B**) TEWL of CPF-gel and CPF-AZ-gel, respectively, expressed as g/h × m^2^. (**C**,**D**) the SCH of CPF-gel and CPF-AZ-gel respectively expressed as arbitrary units (AU). Significant statistical differences: * *p* < 0.05, ** *p* < 0.01, *** *p* < 0.001, **** *p* < 0.0001, ns = non-significant. (**E**) Comparison of the evolution of the TEWL between both formulas: CPF-gel and CPF-AZ-gel. (**F**) Comparison of the evolution of the SCH between both formulas: CPF-gel and CPF-AZ-gel. Each value represents the mean ± SD (n = 10).

Figure 13 shows the progression of the monitored parameters (TEWL and SCH) before and after the application of the formulations for up to 120 min. These parameters are indicative of the effect of the formulations on skin hydration and integrity. The transepidermal water loss (TEWL) values obtained from CPF-gel and CPF-AZ-gel showed a slight increase 5 min after the application and then a decrease at 30 min, and finally, remaining unchanged, being greater than the values for the formulation CPF-AZ-gel (Figure 13A,B,E). The stratum corneum hydration (SCH) values obtained from CPF-AZ-gel presented a slight increase 5 min after the application, with this increase being higher than the one for the CPF-gel formulation, and then this descended quickly; this occurs because the water in the formulas evaporates after 30 min and then both formulations remain unchanged, reaching values lower than the basal value (Figure 13C,D,F). Considering that skin hydration is directly related to skin capacitance, the results suggest that the formulations slightly increase hydration in relation to the normal behavior of the skin. No visible skin irritation was observed after the formulations were applied to the skin of the patients, indicating that both formulations were well tolerated on the skin.

### 2.11. Antimicrobial Efficacy

Caspofungin belongs to an essential class of antifungals, the echinocandins, which are used to treat invasive candidiasis that is resistant to conventional treatments. Candidiasis is caused mainly by *C. albicans*, *C. glabrata*, *C. parapsilosis*, and *C. tropicalis* [48]. In this study, in vitro tests were carried out to determine if the Candida strains tested were susceptible to the formulations under investigation.

**Figure 14:**
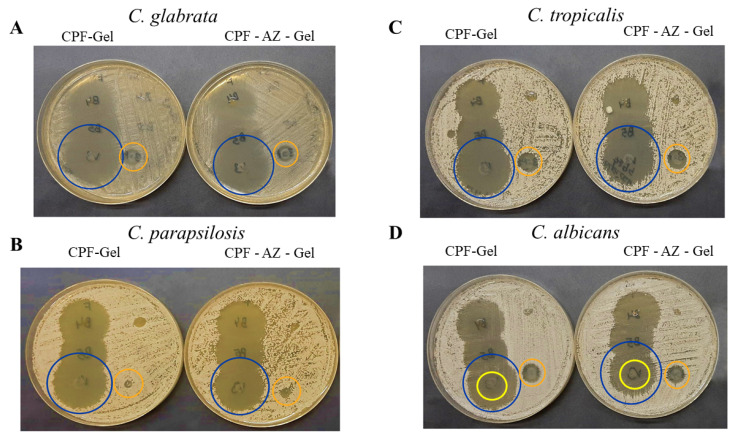
In vitro antifungal activity. (**A**) *C. glabrata* (**B**) *C. parapsilosis* (**C**) *C. tropicalis* and (**D**) *C. albicans*. The blue circles show the inhibition halo for CPF-gel and CPF-AZ-gel, the orange circles show the halo growth around the disc with an excipient (gel), and the yellow circles indicate the intrahalo growth of C. albicans.

In this study, the CPF-gel and CPF-AZ-gel formulations demonstrated that they are effective at the in vitro level in three of the four Candida strains tested, producing broad areas of inhibition (Figure 14A–C, yellow circles), which indicates a beneficial effect since resistance to these types of drugs is gaining momentum over time, especially among isolates from immunosuppressed patients [49]. In the case of *C. albicans*, the growth of intrahalo colonies was observed within the zone of inhibition, showing resistance (orange arrow). This behavior has been observed in a previous study [39]. The gel without the drug produced a slight inhibitory effect in the area where the formulations were added. This could be due to the impact caused by chitosan since the latter has been shown to have an antibacterial and antifungal effect (Figure 14A–C, blue circles) [50]. The results are summarized in Table 6.

## 3. Conclusions

Two gels containing caspofungin, which is an echinocandin antifungal, were prepared, CPF-gel and CPF-AZ-gel, with the latter containing Azone and Transcutol-P, which are penetration enhancers. The gels were analyzed for their viscosity and rheological behavior. The spreadability of the gels was also investigated, and all characteristics were suitable for topical administration.

The drug release profile from the gels and the capacity of caspofungin to diffuse through the skin were also evaluated. The release of caspofungin followed a one-phase exponential association model, showing CPF-AZ-gel to have a higher drug amount released. The gel containing Azone and Transcutol-P (CPF-AZ-gel) resulted in a higher retention of the caspofungin in the skin while limiting the diffusion of the drug to the receptor fluid. Hence, a higher amount of caspofungin is available in the skin for a local effect.

Both gels were well-tolerated since they did not show any damage to the skin in the histological study, nor was any irritation observed when the formulation was applied to the skin. A decrease in the TEWL values indicated that the gels did not alter the skin function barrier.

Finally, the antifungal activity was assessed on different *Candida* sp. The two gels inhibited the growth of *C. glabrata*, *C. parapsilosis*, and *C. tropicalis*. However, *C. albicans* showed *resistance*.

In summary, the two gels showed satisfactory properties for a cutaneous application. Caspofungin was located in the skin, especially when applied through CPF-AZ-gel, through which it can exert a local effect. The formulations were well-tolerated, and the gels were effective against three of the four strains tested.

## 4. Materials and Methods

### 4.1. Materials

Caspofungin acetate salt (molecular weight ~1200 Da) was acquired from SunPharma (Barcelona, Spain). Poloxamer 407 (Pluronic F-127, molecular weight ~12,500 Da) was supplied by BASF (Barcelona, Spain), chitosan medium molecular weight (190–310 KDa and deacetylation degree ≥ 75%) was purchased from Sigma Aldrich (Madrid, Spain) and diethylene glycol monoethyl ether (Transcutol-P, molecular weight ~130 Da) was provided by Gattefossé (Saint-Priest, France). Azone (molecular weight ~280 Da) was acquired from Netqem (Durham, NC, USA), and acetic acid and reagents for the analytical method were acquired from Panreac (Barcelona, Spain). Purified and filtered water was obtained using a Milli-Q^®^ Gradient A10 system apparatus (Millipore Iberica SAU.; Madrid, Spain).

### 4.2. Preparation of Gel Formulations

For CPF-gel preparation, Pluronic F-127 and caspofungin were dissolved using cold purified water at 4 °C under magnetic stirring for 30 min, then chitosan previously dissolved in 0.1 M aqueous acetic acid solution was incorporated, maintaining stirring conditions for 20 min. For CPF-AZ-gel preparation, the water proportions of CPF-gel were adjusted to incorporate Azone previously mixed with Transcutol-P.

### 4.3. Thermosensitive Properties

In order to observe if there was a phase transition, the gels were acclimatized in vials at 4 °C, 25 °C, and 32 °C. When the desired temperature was reached, the vials were inclined at approximately 45° to determine if the hydrogels flowed or not. The gels were photographed with and without the drug at different temperatures.

### 4.4. Fourier Transform Infrared

We investigated any possible chemical interaction between caspofungin and the polymers by Fourier Transform Infrared (FTIR). For this, the gels were desiccated in an oven at 37 °C. The FTIR spectra were obtained by a Nicolet iZ10 (Thermo Scientific, Waltham, MA, USA) with a DTGS detector within the range of 4000–525 cm^−1^ with a spectral resolution of 4 cm^−1^ using attenuated total reflectance (ATR) with a diamond crystal. A total of 32 scans per spectrum were obtained.

### 4.5. Morphological Study and Determination of the Porosity of the Hydrogels

Scanning electron microscopy (SEM) was performed to investigate the gels’ structure. To this end, the gels were desiccated in an oven at the temperature of 37 °C, monitoring the desiccation process: the gels were weighed every day until a constant weight was observed. A small amount of the dried gel was coated with a thin film of carbon to obtain a conductive sample suitable for observation by SEM JSM-7001F (JEOL, Inc, Peabody, MA, USA).

The porosity of the hydrogels (CPF-gel and CPF-AZ-gel) was determined by the density method [REF]. Briefly, weighed amounts of dried gels were placed in Eppendorf with 1 mL of pure ethanol (n = 3 for each hydrogel). The experiment was conducted at 32 °C. At pre-established time points, the Eppendorfs were centrifuged at 3000 rpm for 3 min, the supernatants of ethanol were withdrawn by automated pipette, and the hydrogels were weighed to monitor the increase of weight, which corresponds to the ethanol uptake by the gel. The increase in weight was observed at different time points until a constant weight was obtained. The porosity was calculated according to Equation (2):

(2) $$P=\frac{W_{s}-W_{d}}{\rho \times V_{s}}\times 100$$

where W_d_ is the dried hydrogel’s weight, W_s_ is the swollen hydrogel’s weight, ρ is ethanol’s density, and V_s_ is the volume of the swollen hydrogel determined by a pycnometer.

### 4.6. Rheological Behavior

Determining the rheological behaviour in topical products is essential in topical products because theological properties are related to the consistency, texture and spreadability of the product. The viscosity of the formulation will impact the ease of application of the formulation to the skin. The rheological study was carried out with a Haake Rheostress 1^®^ rheometer (Thermo Fisher Scientific, Karlsruhe, Germany) using a cone-cone system (C60/2°Ti: 60 mm diameter, 2° angle). The shear stress (τ) and the viscosity (η) were determined as a function of the shear rate (γ) at 25 ± 0.1 °C. The temperature was set with a thermostatic circulator Thermo Haake Phoenix II + Haake C25P. The Rotational measurements involved a 3-phase program which consisted of a ramp-up shear rate from 0 to 50 s^−1^ for 3 min, followed by a steady shear rate at 50 s^−1^ for 1 min and finally, a ramp-down from 50 to 0 s^−1^ for another period of 3 min. The viscosity was calculated at a steady shear at 100 s^−1^.

The obtained data were analyzed with Data Manager v. 4.87 software (Haake Rheowin^®^, Thermo Electron Corporation, Karlsruhe, Germany) Data from the flow curves were modelled to different mathematical models [51]; and the best-fit model was selected on the basis of correlation coefficient and chi-square value.

### 4.7. Spreadability Analysis

Extensibility testing was carried out by placing a sample of 0.5 g of each formulation (CPF-gel and CPF-AZ-gel) between two glass plates, the one located in the bottom position pre-marked. The standard weight was added onto the upper plate for 60 s forcing the sample to spread. Nine pieces of increasing standard weight (10, 20, 30, 40, 50, 100, 150, 200, and 250 g) were added successively with 60 s between weights. The increase in the spreading area (diameter) was recorded as a function of the weight applied. The spreading area at each applied mass was calculated according to the following equation:

(3) $$S=d^{2}\times \frac{\pi }{4}$$

in which *S* is the spreading area (cm^2^) calculated from the applied mass (g), and *d* is the mean diameter (cm) reached by the sample [39].

The formulations were analyzed in accordance with the best kinetic model, and the extensibility data were fitted to different mathematical equations (hyperbola, Boltzmann) using GraphPad Prism^®^ version 8.0.0 for Windows, GraphPad Software, San Diego, CA, USA. The model fitting appropriateness was confirmed by the r value.

### 4.8. Evaluation of the Drug Release Kinetics

The rate and extent that caspofungin was released from the formulations was evaluated by Franz cells, which consist of two compartments: the donor and receiver chambers. The latter was filled with saline solution as the receptor medium, and the system was kept at 32 ± 1 °C with constant stirring. These experimental conditions provided the sink conditions. The two compartments are divided by a membrane that acts as an inert support for the formulation. The membrane used in this study was polytetrafluoroethylene (PTFE) 47 mm in diameter and 0.45 µm pore size (Merck, Spain). An amount og 0.3 g of formulation for either CPF-gel or CPF-AZ-gel was applied to the membrane in the donor chamber, and the drug diffused through the membrane into the receiver compartment was assessed over time by collecting samples (200 µL) at the following time points: 1, 2, 4, 6, 22, 26, and 28 h. The samples were analyzed by UPLC. 6 replicates for each formulation were included in this study.

The cumulative amount of caspofungin released was calculated and plotted as a function of time. The release rate was calculated by linear regression analysis as the slope of the linear part in the release profile. Kinetic modeling was performed to describe the behavior of caspofungin release over time. To this end, the data were fitted to several mathematical models, and the best-fitted one was chosen on the basis of the coefficient of determination (R^2^).

### 4.9. Evaluation of the Permeation Capacity of Caspofungin through Ex Vivo Human Skin

To assess the potential of caspofungin to be absorbed into the bloodstream and determine the effectiveness of the gels developed as topical delivery systems, an ex vivo permeation test was conducted by Franz cells. The set-up is similar to the drug release test, with the particularity of using ex vivo human skin as a membrane. The skin was obtained from the abdominal area of donors subjected to aesthetic surgery; the “Docencia e Investigación” Committee of SCIAS Hospital de Barcelona approved the study (approval date 17 January 2020). The skin was cut at the thickness of 0.4 mm by a dermatome (Dermalab GA630 dermatome, Aesculap, Tuttlingen, Germany). This thickness presents the main layers of the skin: stratum corneum, viable epidermis, and a representative part of the dermis, which contains the blood vessels. The integrity of the skin discs was evaluated before the permeation test by measuring the transepidermal water loss (TEWL). Briefly, the sensor was placed on the skin for 2 min to allow it for temperature and humidity equilibration. Afterward, the TEWL values were recorded for 20 s, and only skin discs with TEWL below 13 g/h·cm^2^ were included in the study [43].

The permeation test was performed under an infinite dose approach, and 0.3 g of gel (CPF-gel or CPF-AZ-gel) was applied to the skin on the stratum corneum side, and samples were collected from the receiver medium at the time points 0.5, 1, 2, 4, 6, 22, 26 and 28 h. Samples were analyzed by UPLC to estimate the amount of caspofungin that permeated through the skin over time. Six replicates per formulation were included in the study. Once finished the permeation tests, 3 skin discs per formulation were destined for drug extraction, and the other 3 replicates were used for a histological evaluation.

To determine the amount of caspofungin that remained in the skin after 28 h of permeation, the skin discs were processed for drug extraction as follows: firstly, the excess gel on the skin surface was gently wiped and rinsed with distilled water; then, the diffusional area exposed to the formulations (2.54 cm^2^) was cut, punched, and immersed in distilled water. The drug was extracted by ultrasonic technique for 20 min, and samples were analyzed by UPLC.

#### Data Analysis

The cumulative amounts of permeated caspofungin were plotted for each sampling time point. To evaluate the rate and extent of caspofungin permeation, we calculated the following permeation parameters: flux (J) as the permeation rate, expressed in µg/h; the lag-time (Tl), the permeability coefficient (K_P_), and the partition and diffusion coefficients (P1 and P2, respectively) were also estimated, as well as we predicted the plasma concentration at the steady state that caspofungin would achieve after a topical application of the formulations, CPF-gel or CPF-AZ-gel, in a theoretical surface of application of 5 cm^2^ [52].

The amount of caspofungin retained in the skin after 28 h of exposure was calculated by extracting the drug from the tissue and applying the factor of drug recovery in the skin of 45.5%.

### 4.10. Analytical Method for Determining Caspofungin

The samples from the drug release and permeation studies were analyzed by Ultra Performance Liquid Chromatography (UPLC) using an Acquity I-Class UPLC System (Waters, Milford, CT, USA). For the determination of caspofungin, we used a Lichrospher RP-8 column (125 × 4 mm, 5 µm, Phenomenex) in gradient elution, which consisted of 50% B for 0–5 min; 100% B for 5–6 min and finally, 50% B for 6–8 min, being the composition of the mobile phase: A = 0.1 % of trifluoroacetic acid (TFA), and B = methanol. The flow rate was set at 0.8 mL/min, and we injected a sample volume of 10 µL. The detection of caspofungin was carried out by an Acquity Fluorescence Detector at the excitation wavelength of 224 nm and emission wavelength of 304 nm [39].

### 4.11. Histological Analysis of the Ex Vivo Human Skin after Permeation of Caspofungin

The histological analysis aimed to examine the skin tissues to evaluate whether any alteration in the skin structure had occurred after the permeation study. To this end, 3 skin discs per formulation from the ex vivo permeation test were processed for the histological examination. After treatment with the different formulations, the skin was rinsed in PBS, dehydrated, and finally embedded in paraffin wax. Tissue sections of 5 µm were stained with hematoxylin and eosin to assess the impact of the different formulations on the skin layers. Serum was used as a control condition, and ethanol was used as a positive control. Finally, the skin samples were observed under the microscope (Olympus BX41) and photographed (camera Olympus XC50).

### 4.12. Tolerance Studies by Evaluating Biomechanical Skin Properties

An in vivo skin tolerance study was conducted to evaluate the biomechanical properties of human skin. The study protocol was approved by the Ethics Committee of the University of Barcelona on 30/01/2019 (IRB00003099). The assessment of the total amount of water loss (TEWL) through the skin was carried out using a Tewameter^®^ TM 300 (Courage-Khazaka Electronics GmbH, Cologne, Germany) to measure the amount of water reaching the surrounding atmosphere through the diffusion and evaporation processes of the epidermal layer of the skin. Ten healthy-skinned participants ranging in age from 25 to 40 years were recruited after medical assessment and notification (written informed consent) of the nature of the study and associated procedures. The subjects were asked not to use skin-care cosmetics on the measurement site (flexor side of the left forearm) during the day before the study. The volunteers stayed in the testing room for at least 20 min before taking the measurements. The measurement site was marked drawing circles around 4 cm in diameter The readings were collected (baseline readings), and then we applied a uniform layer of 0.5 g of the formulations (CPF-gel and CPF-AZ-gel) to the center of the circle using a gentle tool using a circular motion with the thumb to help distribute samples. A total of 20 laps were carried out in a clockwise direction. New measurements were collected just after application and at 30, 60, 90, and 120 min post-application [31]. To measure, the electrode, a small hollow cylinder, was maintained on the different tissue’s surface for 1 min. TEWL values (g/m^2^·h) were expressed as the mean ± SD of 10 replicates before and after the application of the formulations for at least 2 h. All measurements were carried out in accordance with published procedures [53,54].

The measurement of the hydration of the stratum corneum (SCH) was determined before application in the basal state and 5, 30, 60, 90, and 120 min post-applications of CPF-gel and CPF-AZ-gel on the treated area using Corneometer^®^ 825 a Multi Probe installed Hydration Adapter^®^ MPA5 (Courage & Khazaka Electronics GmbH, Cologne, Germany). The measurements were performed using the capacitance method, which takes advantage of the relatively high dielectric constant of water compared to other skin substances. Stratum corneum hydration (SCH) values (arbitrary units, AU) are expressed as mean ± SD, n = 10.

### 4.13. Antimicrobial Efficacy

The in vitro antifungal activity of the prepared formulations was determined using the methodology described in the test protocol [citation from CLSI], similar to the disk diffusion method (also known as the Kirby-Bauer method) but with some modifications.

The *Candida* strains used in this assay were: *Candida albicans* ATCC 10231, *Candida glabrata* ATCC 66032, *Candida parapsilosis* ATCC 22019, and *Candida tropicalis* ATCC 7349 (American Type Culture Collection, Manassas, VA, USA).

For the inoculum preparation, the culture medium was first prepared; Muller Hinton Agar (MH) supplemented with 2% glucose (MH-Glucose 2%) and 500 µg/mL of chloramphenicol to avoid possible bacterial contamination by the excipients and incubating at 30 °C for 48 h. Subsequently, each of the *Candida* strains was seeded. The fungal inoculum was prepared by suspending one to two isolated yeast colonies in Ringer’s solution to achieve 0.5 McFarland equivalent density.

The 2% MH-glucose plates were inoculated three times over the entire surface of the agar with the aid of a swab soaked in the yeast with the scratching action, rotating the plate approximately 60° each time to ensure a good distribution of the inoculum.

The following formulations were studied: CPF-gel, CPF-AZ-gel, gel (excipient), and controls of 100 UI/mL nystatin and 250 µg/mL amphotericin B. Approximately 5 µL of these products were placed in the inoculated yeast and incubated at 30 °C for 48 h. The inhibition zone for yeast growth was observed.

## Figures and Tables

**Table 1:** Composition formula of caspofungin-loaded gels.

Ingredients	CPF-Gel (% *w*/*w*)	CPF-AZ-Gel (% *w*/*w*)
Caspofungin	2	2
Chitosan	2	2
Pluronic F-127	18	18
0.1 M Acid acetic solution	20	20
Water	58	48
Azone	-	5
Transcutol-P	-	5

**Table 2:** Porosity of caspofungin-loaded gels.

Formulation	Porosity (%)
CPF-gel	5.7 ± 0.3
CPF-AZ-gel	22.4 ± 1.4

**Table 3:** Viscosity and mathematical fitting of caspofungin formulations at 10, 25 and 32 °C.

Temperature Conditions		10 °C	25 °C	32 °C
CPF-gel	Viscosity (Pa·s) at 100 s^−1^	1.48 ± 0.01	1.16 ± 0.01	0.95 ± 0.03
	Mathematical model (stretch ramp up/down)	Cross	Cross	Cross
CPF-AZ-gel	Viscosity (Pa·s) at 100 s^−1^	1.66 ± 0.02	1.52 ± 0.01	1.09 ± 0.01
	Mathematical model (stretch ramp up/down)	Cross	Cross	Cross

**Table 4:** Fitting and goodness of fit for the parameters estimated according to one-site hyperbola equations for the formulas CPF-gel and CPF-AZ-gel.

One Site Binding (Hyperbola)	CPF-Gel	CPF-AZ-Gel
Best-fit values		
Bmax	36.23	39.41
Kd	13.98	11.50
Std. Error		
Bmax	0.9566	1.008
Kd	1.719	1.500
95% CI (profile likelihood)		
Bmax	34.29 to 38.29	37.37 to 41.57
Kd	10.67 to 17.92	8.612 to 14.92
Goodness of Fit		
Degrees of Freedom	25	25
R squared	0.8725	0.8480
Sum of Squares	142.0	176.0
Sy.x	2.383	2.653
AICc	51.86	57.66

**Table 5:** Permeation parameters estimated for CPF-gel and CPF-AZ-gel.

Parameter	CPF-Gel	CPF-AZ-Gel
A_28h_ (µg)	1854.4 ± 193.2	1623.9 ± 173.2
J (µg/h)	129.2 ± 14.7	86.9 ± 1.3 ***
J/cm^2^ (µg/h/cm^2^)	50.8 ± 5.8	34.2 ± 0.5 ***
Tl (h)	13.9 ± 1.2	9.2 ± 1.4 ***
K_P_ (10^−3^ cm/h)	2.54 ± 0.29	1.71 ± 0.02 ***
P_2_ (10^−2^ 1/h)	1.20 ± 0.14	1.79 ± 0.11 ***
P_1_ (10^−2^ cm)	21.23 ± 0.21	9.53 ± 0.02 ***
Css (ng/mL)	26.99 ± 3.08	18.16 ± 2.65 **

A_28h_: cumulative amount of caspofungin permeated at 28 h. J: flux; Tl: lag-time; K_P_: permeability coefficient; P_1_: partition coefficient vehicle-skin; P_2_: diffusion coefficient and Css: predicted plasma concentration at the steady state. ** statistical significance *p* < 0.01 and *** statistical significance *p* < 0.0001.

**Table 6:** Growth inhibition on *Candida* species.

Yeast Tested	CPF-Gel	CPF-AZ-Gel	Gel
*C. albicans*	R^(a)^	R^(a)^	R^(b)^
*C. glabrata*	S	S	R^(b)^
*C. parapsilosis*	S	S	R^(b)^
*C. tropicalis*	S	S	R^(b)^

S: susceptible, R^(a)^: resistant to inhibition of halo and intrahalo colonies, and R^(b)^: resistant, with the formation of a slight halo of inhibition.

## Data Availability

Data is contained within the article.
